# Suppression of pseudogene MT2P1 transcription induced by E2F7 inhibits hepatocellular carcinoma cell proliferation and facilitates apoptosis via preserving its parental gene

**DOI:** 10.1080/15384047.2025.2510035

**Published:** 2025-05-23

**Authors:** Yiquan Lu, Yifan Zhang, Fengjie Hao, Nan Wang, Yongjun Chen, Junqing Wang

**Affiliations:** a Department of General Surgery, Ruijin Hospital, Shanghai Jiao Tong University School of Medicine, Shanghai, People’s Republic of China; b Department of Internal Medicine III, University Hospital RWTH Aachen, Aachen, Germany

**Keywords:** Pseudogene, MT2P1, miR-15b-5p, MT2A, hepatocellular carcinoma

## Abstract

The majority of the pseudogenes are inert in normal transcription. Their transcripts are mostly attributed to non-coding RNAs that play various functions in human tumorigenicity and progression. Distinctively, pseudogene MT2P1 is universally transcribed in hepatocytes and presents a significant decrease in hepatocellular carcinoma (HCC). The effect of MT2P1-RNA on HCC cell proliferation and apoptosis needs investigation. MT2P1-RNA was detected by RT-qPCR assay in HCC tissues and cell lines, combined with the exploration of the public databases. The immunohistochemistry assay was used for testing the expression profile of E2F7 and the parental gene MT2A. The clinicopathological features of the patients were collected and analyzed. Ectopic expression of MT2P1-RNA in HCC cell lines was conducted, and the CCK8 assay and flow cytometry assay were carried out. Chromatin immunoprecipitation assay and Dual-luciferase reporter assay were, respectively, applied to validate the interaction between MT2P1, E2F7, and microRNA-15b-5p. The downregulation of MT2P1-RNA in HCC is negatively correlated with dismal clinicopathological features. MT2P1-RNA significantly suppressed HCC cell proliferation and induced apoptosis. E2F7 depletion sequentially elevated the level of MT2P1-RNA and MT2A, and E2F7 was validated as a suppressive transcription factor of the MT2P1 gene. The direct interactions of either MT2P1/miR-15b-5p or miR-15b-5p/MT2A were, respectively, ascertained, enlightening the ceRNA effect of them. The pseudogene-derived MT2P1-RNA is a suppressor of HCC by exerting the ceRNA effect on preserving MT2A, and its transcription is regulated by the suppressive transcription factor E2F7.

## Introduction

Patients with hepatocellular carcinoma (HCC) suffer severe tumor invasion, metastasis, and poor outcomes along with high mortality, due to the potent tumor heterogeneity and aggressive cell biological behaviors.^1–3^ The discovery of innovative and practical targets for HCC prevention and treatment is of importance to researchers.

Tremendous DNA transcripts are generated from the human genome, some of which lack protein-coding capability and are called non-coding RNAs (ncRNAs). ncRNAs are classified according to their length. Long non-coding RNAs (lncRNAs) generally consist of over 200 nucleotides and present much more complex functions and mechanisms in regulating tumorigenesis and development, most of which remain unknown to researchers.^4,5^

Pseudogenes are DNA sequences that lack a promoter, have premature stop codon or frameshift mutated, generated through mutation and duplication from the parental protein-coding genes.^6–8^ Pseudogenes are limited in transcription, while the pathological activation of pseudogenes has been discovered to generate transcriptions, usually regarded as lncRNAs with various functions.^9^ Taking advantage of the complex functions of the lncRNAs, we make sense of their functions through the lncRNA-related functions to a large extent, for example, the miRNA decoy effect, or so-called competitive endogenous RNA (ceRNA) effect.

The ceRNA effect suggests that certain ncRNA have high affinity for some miRNAs, competing with mRNA for those miRNA’s specific binding sites which protects mRNA from miRNA-mediated degradation.^10,11^ For pseudogene-derived transcripts, the ceRNA effect has been gradually validated as one of the main mechanisms in tumor progression and caught the attention of discovering new targets for tumor research. For example, the transcript of pseudogene PTENP1 efficiently binds to miR-21 and miR-19, which are considered oncogenes, respectively, in gastric cancer and clear cell renal carcinoma by post-translationally degrading the parental gene PTEN.^12,13^ In HCC, pseudogene AKR1B10P1 transcript has been verified in our recent research that acts as a molecular sponge in a ceRNA way to enhance tumor growth by competitively binding to miR-138.^14^

We constructed an online database and an analysis tool for the Liver Cancer lncRNA Explore (LCLE) to present the expression profile of lncRNAs and the related network in HCC (https://datasciences123.shinyapps.io/LCLE/).^15^ By applying this platform, we have screened a series of pseudogene-derived transcripts that are differently expressed in HCC. As our team discovered, the majority of pathological pseudogenes in HCC are almost specifically expressed in HCC tissues, barely transcribed in normal tissues, such as UBE2BP1^16^ and SNRPFP1,^17^ and correlated with poor prognosis of patients and acceleration of tumor development. However, on the opposite, Metallothionein 2 Pseudogene 1 (MT2P1) is highly expressed in normal liver tissues and potently suppressed in HCC, negatively correlated with poor outcomes in HCC patients. In *vitro*, experiments introducing MT2P1 in HCC cells impaired the capacity of cell proliferation and induced cell apoptosis.

MT2P1 is a processed pseudogene derived from Metallothionein 2A (MT2A).^18^ In human malignancies, MT2A presents pendulous functions depending on different tumor types. We noticed a significant positive correlation between MT2P1 and MT2A, and the level of MT2A in HCC cells was significantly elevated by ectopically expressing MT2P1, which implicated a possible mechanism in HCC development.

Members of the E2F Transcription Factor family are up-regulated in most human cancers via complex dysfunction of their upstream modulators. The majority of E2Fs present active transcription functions in the tumor process.^19^ As an exception, E2F7 is classified as the sporadic atypical member depending on the different regulating mechanisms and the inverted repressive regulation of the genes for cell proliferation.^20^ Here, we found that highly expressed E2F7 in HCC cells was correlated with the decline of the transcription of both MT2P1 and its parental gene MT2A. Meanwhile, by depleting E2F7, we confirmed the E2F7’s transcriptional suppression effect on MT2P1, along with the validation of the interaction between MT2P1-RNA and E2F7. The MT2P1-RNA change induced by E2F7 was followed by the expression modulation of MT2A in the same trend. Combined with online prediction and the following dual-luciferase reporter assay, we validated a ceRNA effect of MT2P1-RNA on miR-15b-5p, targeting MT2A as an oncogene. Thus, we ascertained the intermediary role of MT2P1 in HCC cell growth by preserving its parental gene MT2A in a ceRNA way, and under the control of the suppressive transcriptional regulation of E2F7. Our findings in this study could provide the possibility to find innovative targets for HCC control.

## Results

### MT2P1-RNA was potently down-regulated in HCC cell lines and tissues

**Figure 1.**
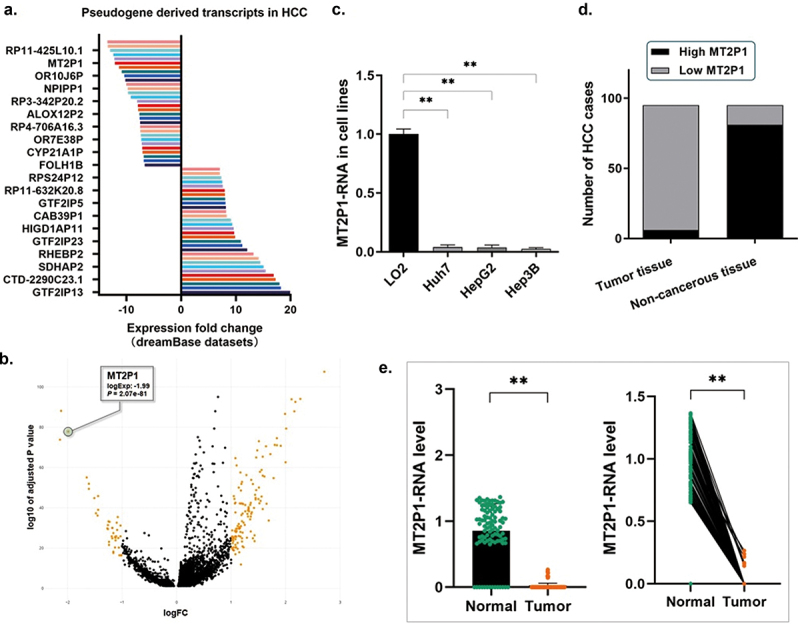
MT2P1-RNA was down-regulated in HCC cell lines and tissues.

The dataset analysis of both the dreamBase and LCLE databases presented high MT2P1-RNA levels in the normal liver tissues and negligible transcription levels in the HCC tumor tissues (Figure 1a,b). In the three recruited HCC cell lines (Huh7, HepG2, and Hep3B), MT2P1-RNA was detected at an extremely low, almost undetectable level, compared with the control LO2 cells (Figure 1c).

(a) The dataset analysis of the dreamBase presented a high MT2P1-RNA level in the normal liver tissues and was barely transcribed in the HCC tumor tissues. (b) The dataset analysis of LCLE databases demonstrated a significantly lower expression of MT2P1-RNA in HCC tumor tissues than in the non-cancerous liver tissues. (c) RT-qPCR assay demonstrated a significant decrease of MT2P1-RNA in the three recruited HCC cell lines (Huh7, HepG2, and Hep3B), in comparison with the control LO2 cells (**P < 0.01). (d) Statistic of the number of cases concerning the expression of MT2P1-RNA in HCC specimens (*n* = 95). A significant decrease of MT2P1-RNA in most of the HCC tissues, and only a small portion of the tumor tissues (6.32%, 6/95) presented the detectable MT2P1-RNA at a low level. (e). RT-qPCR assay was conducted on the 95 real patients’ specimens. The MT2P1-RNA was highly expressed in the non-cancerous liver tissues, and only very few detectable UBE2MP1 transcripts were found in the non-cancerous tissues (**P < 0.01).

The detection of the patients’ specimens collected from our medical center also demonstrated a significant decrease of MT2P1-RNA in most of the HCC tissues, and only a small portion of the tumor tissues (6.32%, 6/95) presented the detectable MT2P1-RNA. While a relatively higher MT2P1-RNA expression was detected in most of the non-cancerous tissues (85.26%, 81/95) (Figure 1d,e).

According to the analysis of the online databases, the parental gene MT2A was also significantly decreased in HCC tumor tissues and was positively correlated with the pseudogene-derived MT2P1-RNA (Supplementary Figure S1a,b). The decrease of MT2A in HCC tissues was further demonstrated by using the RT-qPCR and IHC assays on patient samples and cell lines (Supplementary Figure S1c,d).

### The decrease of MT2P1-RNA is correlated with unsatisfactory clinicopathologic features in HCC patients

The correlation between the MT2P1-RNA level and the clinicopathologic features of the 95 hCC patients was statistically analyzed. As demonstrated in Table 1, no significant correlation between MT2P1-RNA and the patient’s age, gender, tumor size, and virus control status was observed. While the lower expression of MT2P1-RNA is positively related to a higher quantity of serum Alpha-fetoprotein (AFP) (*p* < .05), more advanced TNM stages (*p* < .05), more tumor microsatellite formation (*p* < .05), detectable invasion of venous (*p* < .05), and later liver cirrhosis stages (*p* < .05). These findings prompt that MT2P1-RNA might play a protective role in HCC development.

**Table 1.** Correlation between MT2P1-RNA and clinicopathological features in 95 hCC specimens.

Clinicopathologic parameters	MT2P1		P*
	Undetectable (*n* = 89)	Detectable (*n* = 6)	
Age (years) ≤50 <50			
	56	3	0.670
	33	3	
Gender Male Female			
	47	4	0.684
	41	2	
Diameter (cm) ≤5 <5			
	41	5	0.104
	48	1	
TNM stage I～II III～IV			
	22	4	0.046
	67	2	
Tumor encapsulation Absent Present			
	33	3	0.670
	56	3	
Tumor microsatellite formation Absent Present			
	32	5	0.032
	57	1	
Venous invasion No Yes			
	28	4	0.175
	61	2	
HBsAg Negative Positive			
	9	2	0.142
	80	4	
AFP(ng/ml) ≤400 ＞400			
	15	5	＜0.001
	74	1	
Cirrhosis Absent Present			
	8	2	0.119
	81	4	

*P < 0.05.

MT2P1 level associated with clinicopathologic features in 95 hCC patients, including age, gender, tumor size, tumor stage (AJCC), tumor encapsulation, tumor microsatellite formation, vein invasion, HBsAg status, AFP level, and liver cirrhosis. Statistically, significance was assessed by Fisher’s exact text.

### Ectopically introducing MT2P1-RNA impairs HCC cell proliferation and induces cell apoptosis

**Figure 2.**
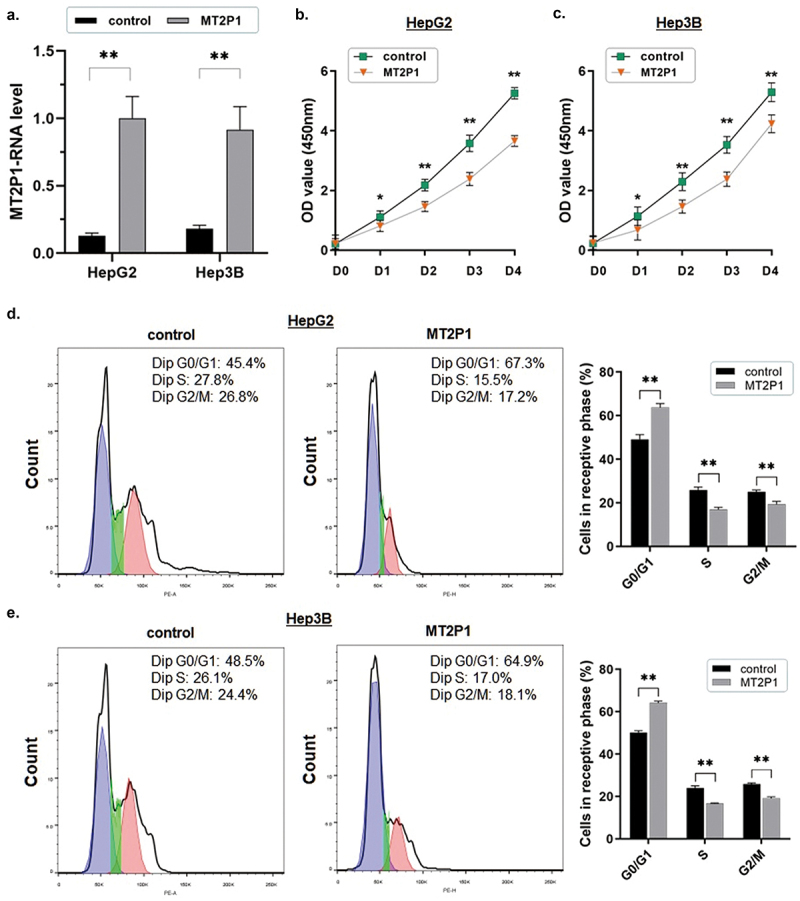
Ectopically introducing MT2P1-RNA impairs HCC cell proliferation and induces cell apoptosis.

The ectopic up-regulation of MT2P1-RNA expression was confirmed by RT-qPCR assay in both HepG2 and Hep3B cells (Figure 2a). The following in *vitro* experiments indicated that the cell proliferation ability in HCC cells was remarkably repressed in these two cell lines when MT2P1-RNA was up-regulated (*P < 0.05; **P < 0.01) (Figure 2b,c). Consistent with this, a significant cell cycle arrest at the G0/G1 phases in the HCC cells was detected by the flow cytometric analysis when MT2P1-RNA was elevated (Figure 2d,e). The percentage of the HepG2 and Hep3B cells in the G0/G1 phase increased from 49.1% to 63.8% (*P* < 0.01) and from 50.2% to 64.2% (*P* < 0.01), respectively. Meanwhile, the percentage of the cells in the S phase declined (HepG2: from 25.9% to 17.0%, *P* < 0.05; Hep3B: from 25.1% to 19.2%, *P* < 0.01), as well as the cells in the G2/M phase (HepG2: from 23.9% to 16.7%, *P* < 0.01; Hep3B: from 25.8% to 19.1%, *P* < 0.01).

(a) MT2P1-RNA was ectopically up-regulated in both HepG2 and Hep3B cells (both repeated three times) and validated using the RT-qPCR assay (**P < 0.01). (b) The CCK8 assay was applied to investigate the cell proliferation ability. Cell proliferation in HepG2 cells was significantly blocked after ectopically up-regulating MT2P1-RNA (*P<0.05, **P<0.01). The assay was repeated three times. (c) The CCK8 assay was also applied to Hep3B cells. Cell proliferation of HepG2 cells was significantly blocked after ectopically up-regulating MT2P1-RNA (*P < 0.05, **P < 0.01). (d) The representative histograms describe the cell cycle profiles of HepG2 cells using flow cytometry are presented. The cell cycle of HepG2 cells was arrested in the G0/G1 phase by up-regulating MT2P1. The results are means of three independent experiments±SD (**P<0.01). (e) HepG2 cells presented similar cell cycle profiles, that the cell cycle was arrested in the G0/G1 phase after MT2P1 up-regulation. The results are means of three independent experiments±SD. (**P < 0.01).

**Figure 3.**
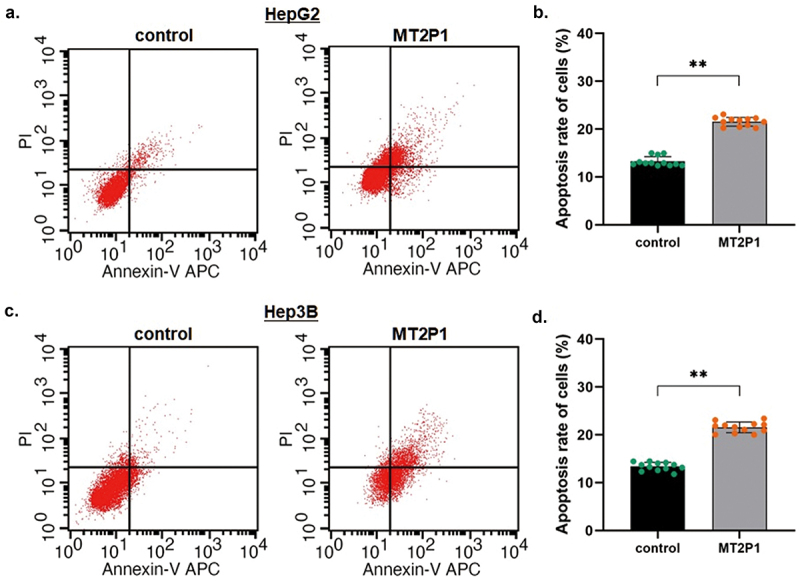
Up-regulating MT2P1-RNA induced HCC cell apoptosis.

In contrast, the flow cytometric analysis showed significantly elevated apoptosis in both HepG2 and Hep3B cells (HepG2: from 13.25% to 25.54%, *P* < 0.01; Hep3B: from 13.39% to 21.56%, *P* < 0.01) induced by introducing MT2P1-RNA. All these findings strongly illustrated that the MT2P1-RNA exerts a potential anti-cancer function by influencing cell growth and maintenance in HCC (Figure 3a–d).

The flow cytometric analysis was conducted to detect the status of cell apoptosis (a～b) The calculated apoptotic HCC cells were significantly increased by the flow cytometric analysis in HepG2 cells (from 13.25% to 25.54%, *P* < 0.01), sequentially induced by introducing MT2P1-RNA. (c～d) The apoptotic HCC cells were increased in Hep3B cells (from 13.39% to 21.56%, *P* < 0.01) by up-regulating MT2P1-RNA.

### E2F7 suppresses MT2P1 transcription in HCC cells

The level of E2F7 expression in HCC was detected from online databases, the HCC tissues, and cell lines. Compared with the non-cancerous liver tissues and cells, a potent up-regulation of E2F7 in HCC was demonstrated (Supplementary Figure S2). Considering E2F7 is a repressive transcription factor, we wondered if the transcription of MT2P1 was modulated by E2F7.

**Figure 4.**
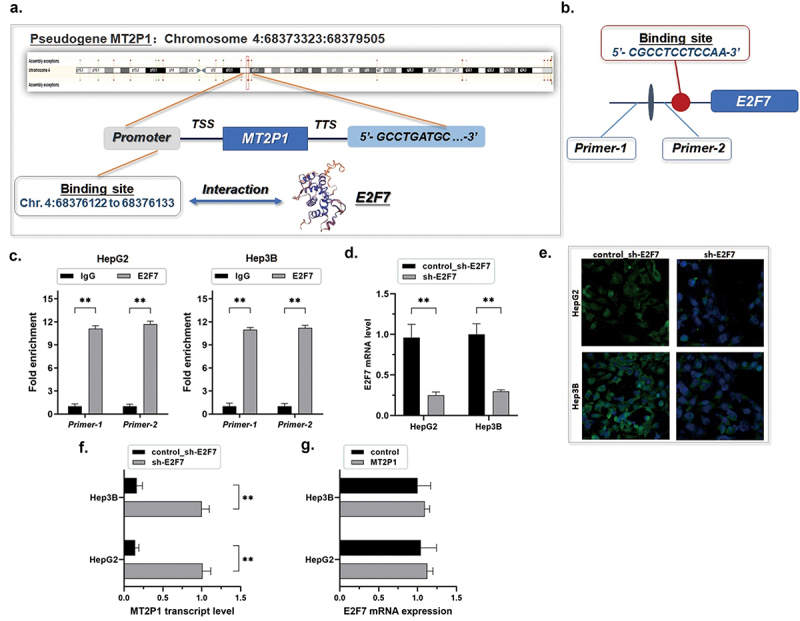
E2F7 suppresses MT2P1 transcription in HCC cells.

We selected a 3000 bp fragment from the upstream sequence of the MT2P1 gene to predict high-affinity binding sites in the transcriptional process. The results were obtained and screened from the Database of Human Transcription Factor Targets (http://bioinfo.life.hust.edu.cn/hTFtarget#!/.) and the Gene-Cloud of Biotechnology Information (GCBI, https://www.gcbi.com.cn). We discovered a potential binding site between E2F7 and an equivalent region of the promoter upstream of the MT2P1 gene (5’- CGCCTCCTCCAA-3’, Chr. 4:68376122 to 68,376,133; *p* = 1.87e-05) (Figure 4a). The definite interaction between E2F7 and the MT2P1 gene was further defined by using the ChIP assay (Figure 4b,c).

(a) E2F7 is a potential transcription factor that binds to the promoter region of the pseudogene MT2P1 (5’- CGCCTCCTCCAA-3’, Chr. 4:68376122 to 68,376,133; *p* = 1.87e-05). (b～c) ChIP assay was carried out to investigate the direct interaction between E2F7 and the equivalent region of the promoter upstream of the MT2P1 gene (**P < 0.01). IgG was used as the negative control. Three biological repeats were done in both groups. (d) Lentiviral vectors containing shRNA were used for depleting E2F7 in both Hep3G and Hep3B cells, and the effect was validated using the RT-qPCR assay and the immunofluorescence detection (**P < 0.01). Triplicate experiments were performed. (e) As the RT-qPCR assay demonstrated, the expression level of MT2P1-RNA consequentially declined with significance after E2F7 depletion (**P < 0.01). (f) Knockdown of E2F7 significantly increases MT2P1 expression (**P < 0.01). (g). There was no significant change observed in E2F7 expression by the RT-qPCR assay when MT2P1-RNA was up-regulated (repeated three times).

Furthermore, we used lentiviral vectors containing shRNA for transfection and significantly depleted E2F7 in both Hep3G and Hep3B cells (Figure 4d,e). E2F7 depletion resulted in a significant consequential increase in the MT2P1-RNA expression (Figure 4f). On the contrary, no significant change in E2F7 expression was observed by RT-qPCR assay when MT2P1-RNA was up-regulated (Figure 4g). Thus, we confirmed that the transcription of MT2P1 is negatively modulated under the control of E2F7.

### MT2P1-RNA plays the ceRNA effect to preserve the parental gene MT2A by sponging miR-15b-5p

**Figure 5.**
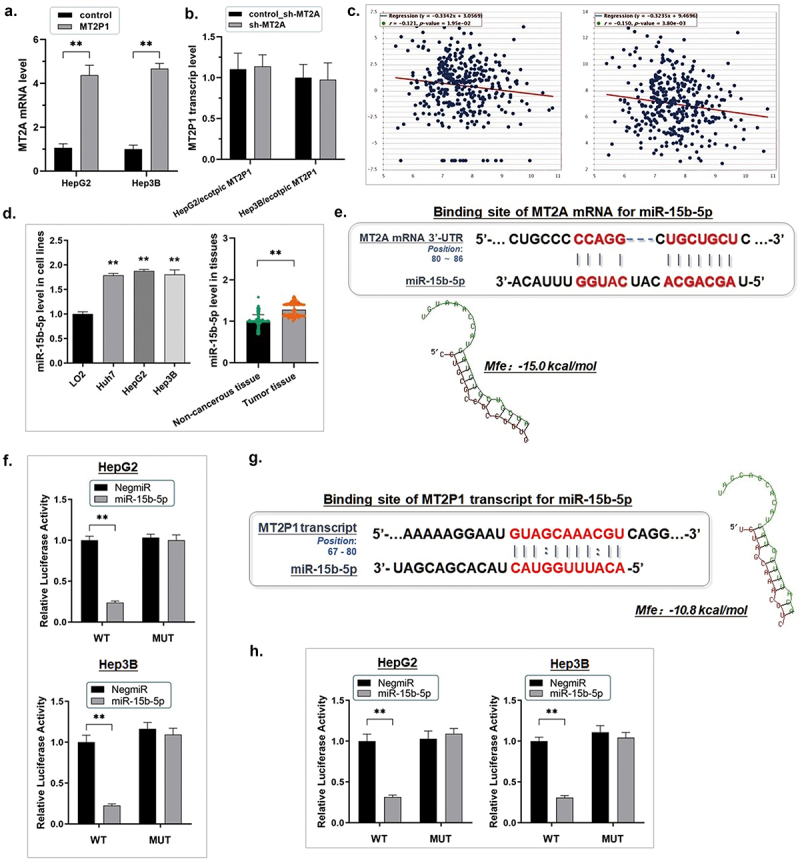
MT2P1-RNA plays the ceRNA effect to preserve the parental gene MT2A by sponging miR-15b-5p.

MT2A mRNA level was consequentially elevated when we ectopically up-regulated MT2P1-RNA in the HCC cells, and no significant expression change of the ectopically expressed MT2P1-RNA was observed by knocking down MT2A (Figure 5a,b). Thus, we supposed MT2P1-RNA stabilized MT2A in some way.

(a) The RT-qPCR assay was carried out. MT2A mRNA level was consequentially elevated when we ectopically up-regulated MT2P1-RNA in the HCC cells (**P < 0.01). Both these and the following experiments were repeated three times. (b) No significant expression change of the ectopically expressed MT2P1-RNA was observed by knocking down MT2A according to the result from the RT-qPCR assay. (c) By exploring the starBase datasets, we noticed that the expression of miR-15B-5p in HCC was negatively correlated with either MT2P1-RNA (*P* = 1.95e-02, *R* = –0.121) or the parental gene MT2A (*P* = 3.80e-03, *R* = –0.150). (d) The RT-qPCR assay demonstrated that miR-15b-5p was highly expressed both in HCC tissues (n = 95) and cell lines (**P < 0.01). (e) The online microcosm (https://www.microcosm.com/) prediction software indicated that miR-15b-5p could bind to the 3’-untranslated region (3’-UTR) of MT2A mRNA (the minimum free energy, Mfe: –15.0 kcal/mol). (f). The dual-luciferase reporter assay was conducted. The signal suppressive effect induced by miR-15b-5p was significantly impaired in the HCC cells transfected with a mutated binding site (**P < 0.01). (g) A short sequence from 67 bp to 80 bp to the 3’ end of the MT2P1-RNA was identified specifically to match the seed region of miR-15b-5p, which prompted a ceRNA effect of MT2P1-RNA on miR-15b-5p (Mfe: -10.8 kcal/mol). (h). The dual-luciferase reporter assay was conducted to validate the direct interaction between miR-15b-5p and MT2P1-RNA. The luciferase signal in both HepG2 and Hep3B cells transfected with miR-15b-5p mimics was decreased, after the transfection of MT2P1/pMIR/WT vectors, compared with the control ones (**P < 0.01). The transfection of MT2P1/pMIR/MUT vectors, on the contrary, did not induce any significant signal change.

By exploring the starBase datasets, we noticed that the expression of miR-15B-5p in HCC was negatively correlated with either MT2P1-RNA or the parental gene MT2A (Figure 5c). Meanwhile, we found that miR-15b-5p was a potential post-transcriptional regulator and mediator connecting MT2P1-RNA and MT2A according to its high expression in HCC tissues and cell lines (Figure 5d). On one hand, the online microcosm prediction software (https://www.microcosm.com/) indicated that miR-15b-5p could bind to the 3’-untranslated region (3’-UTR) of MT2A mRNA (the minimum free energy, Mfe: −15.0 kcal/mol) (Figure 5e). The following dual-luciferase reporter assay demonstrated this direct interaction. In brief, the vectors containing an intercepted sequence from the 3‘UTR of MT2A mRNA (WT-UTR) were selected in the length of 202 bp, and the corresponding control vectors carrying the mutated miR-15b-5p binding site (MUT-UTR) were constructed. Both HepG2 and Hep3B cells were transfected with either of the above two kinds of vectors and also mimicked by miR-15b-5p. Taking the HepG2 cells as an example, the miR-15b-5p mimics (HepG2/miR-15b-5p) defected the luciferase signal of MT2A/pMIR/WT obviously in comparison with the negative control (HepG2/NigmiR). The signal suppressive effect induced by miR-15b-5p was significantly impaired in the cells transfected with a mutated binding site (Figure 5f). Similar results were also demonstrated in the Hep3B cell line.

Simultaneously, as we discovered, a short sequence from 67 bp to 80 bp to the 3’ end of the MT2P1-RNA was identified to specifically match the seed region of miR-15b-5p, which prompted a ceRNA effect of MT2P1-RNA on miR-15b-5p (Mfe: −10.8 kcal/mol) (Figure 5g). We constructed the mutated binding site on MT2P1-RNA to verify the direct interaction between MT2P1-RNA and miR-15b-5p by the dual-luciferase reporter assay. The luciferase signal significantly descended in the two HCC cell lines co-transfected with miR-15b-5p mimics and MT2P1/pMIR/WT vectors, in comparison to the control. Meanwhile, the transfection of MT2P1/pMIR/MUT vectors resulted in no significant signal change (Figure 5h).

### Pseudogene MT2P1 inhibits HCC tumor growth by modulating the post-transcriptional activity of miR-15b-5p on MT2A mRNA degradation

According to the findings described in the above section, it could comprehensively be understood that E2F7 played a suppressive role in MT2P1 transcription and MT2P1-RNA functions as a ceRNA in preserving its parental MT2A by sponging miR-15b-5p. Based on the impairment of cell proliferation and apoptosis resistance by extopical expressing MT2P1-RNA, we observed a significant decrease of miR-15b-5p in HCC cells. Thus, in this section, we further conducted a series of experiments to validate the exact function of this axis on HCC cell growth.

**Figure 6.**
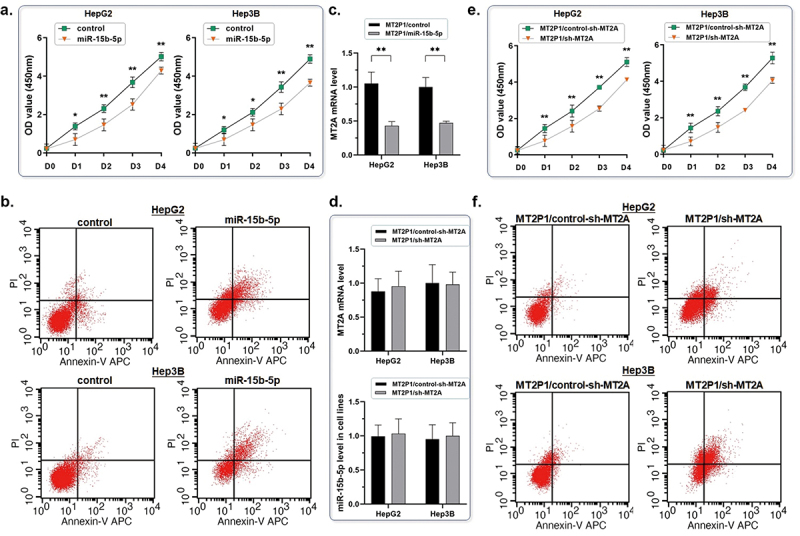
Pseudogene MT2P1 inhibits HCC tumor growth by modulating the post-transcriptional activity of miR-15b-5p on MT2A mRNA degradation.

Firstly, we used mimics to elevate the miR-15b-5p expression again, and the MT2P1-RNA-induced suppression of cell proliferation was recovered along with a significant decrease in the count of the apoptotic cell (Figure 6a,b). Simultaneously, the up-regulation of MT2A induced by MT2P1-RNA was also abrogated when miR-15b-5p was re-elevated (Figure 6c). Secondly, we knocked down the up-regulated MT2A induced by MT2P1-RNA introduction. As expected, the level of either MT2P1-RNA or miR-15b-5p made no change, but the ability of HCC cell proliferation and apoptosis resistance was significantly rescued (Figure 6d–f). According to the above findings, the promotion of HCC cell growth modulated by MT2P1-RNA could be explained by its ceRNA effect on miR-15b-5p through competitively inhibiting the degradation of the terminal effector MT2A in this axis.

(a) The miR-15b-5p expression was elevated again by using the mimics. The MT2P1-RNA-induced suppression of cell proliferation was recovered according to the CCK8 assay (*P < 0.05, **P < 0.01). The experiments were performed three times. (b) The flow cytometric analysis was carried out to present the status of cell apoptosis. The MT2P1-RNA-induced cell apoptosis was reversed when miR-15b-5p was increased. (c) The up-regulation of MT2A induced by MT2P1-RNA was also abrogated when miR-15b-5p was re-elevated (repeated three times, **P < 0.01). (d) The expression of MT2A induced by introducing MT2P1-RNA was knocked down again. The level of either MT2P1-RNA or miR-15b-5p made no change according to all three RT-qPCR assays. (e) The ability of HCC cell proliferation in HCC cells was rescued by knocking down MT2A expression (**P < 0.01). Three samples were tested individually. (f) The increased apoptosis rate of the HCC cells was rescued by knocking down MT2A expression (**P < 0.01) according to the flow cytometric analysis.

## Discussion

Pseudogenes have been regarded as ‘evolution relics’ for a long period.^21^ Whereas, according to recent studies, researchers have gradually discovered the evidence for the potential functions exerted or mediated by pseudogenes in the processes of tumorigenesis and development concerning transcription or post-transcription regulations.^22^

Pseudogenes are not transcribed normally. However, according to the results obtained from the LCLE database, a series of pseudogenes transcriptions are detectable in liver tissues but present significant down-regulation or even no detectable transcription in HCC tissues. This means that the transcriptional characteristics of pseudogenes are not constant. And among the pseudogenes significantly decreased in HCC, we focused on MT2P1, the homologous gene of MT2A. MT2A belongs to the super-family of low-weight intracellular metallothioneins.^23,24^ Innovative evidence has illustrated the differential expression status of MTs in various tumors, and the vital roles of MTs in tumor formation, cell growth, and metastasis.^25^ For MT2A, this isoform of MTs presents a differential expression depending on the type and differentiation status of tumors. In colon cancer cells, MT2A was observed to upregulate and significantly promote cell proliferation and maintenance by inducing chemo-resistance to oxaliplatin.^26^ While in the development of either breast cancer or acute myeloid leukemia, up-regulation of MT2A exerts a remarkable effect on cell cycle arrest and promotes cell apoptosis by influencing the NF-κB pathway.^27,28^ It has been reported that MT2A was down-regulated in HCC and might be used as a prognostic marker for the patients.^29,30^ However, no adequate description or discussion focuses on its pseudogene and the relative regulation mechanisms.

The transcript level of MT2P1 in HCC tissues and cell lines indicated a remarkable down-regulation in comparison with the normal one. We noticed a similar correlation between MT2P1-RNA and its parental gene decrease with the HCC patients’ dismal clinicopathologic features related to unsatisfactory outcomes. The significant decrease of MT2P1-RNA in HCC prompted that its expression was modulated by some transcriptional or post-transcriptional mechanisms during tumor development. The ectopic expression of MT2P1-RNA in HCC demonstrated the potent influence of MT2P1 on the inhibition of cell proliferation and apoptosis resistance. We believe that the positive correlation between MT2P1-RNA and MT2A further implicated the reasonable existence of a modulation concerning HCC growth.

We proceeded with the exploration of the transcription process of MT2P1. Both the online prediction and the following ChIP assay validated the direct interaction between the upstream sequence of the MT2P1 gene and a well-known transcription factor E2F7. E2F7 is an atypical isoform of the E2F family commonly participating in repressive transcriptional modulation in the cell cycle process, and its ambiguous functions result in controversial effects on differential human cancers.^31^ Exceptionally, E2F7 exerts an active transcriptional effect on VEGFa in pancreatic cancer, promoting tumor cell proliferation.^32^ For the HCC, based on the validation of E2F7 over-expression in HCC tissues, we recently found a suppressive transcriptional effect of E2F7 on miR-383-5p and sequentially preserves the downstream SP1/SOX4/Anillin axis, which significantly facilitates tumor growth. These findings strongly prompt the critical regulation function of E2F7 in HCC.^33,34^ In this study, there was no significant change made for E2F7 when we ectopically introduced MT2P1-RNA. We prompt that the recognition of the repressive transcriptional regulation of E2F7 on MT2P1 strongly indicates the mechanism of MT2P1-RNAa decrease in HCC.

We noticed that miR-15b-5p was probably interacting with both MT2A mRNA and MT2P1-RNA. The negative correlation of miR-15b-5p with both MT2P1-RNA and MT2A mRNA further suggested the ceRNA effect. miR-15b-5p has been reported as highly expressed in multiple human malignancies as a promoter of tumor progression.^35,36^ The highly expressed miR-15b-5p was observed in both HCC tissues and cancer cells. Thus, we conducted the dual-luciferase reporter assay and as expected, miR-15b-5p was validated to interact with MT2P1-RNA as a downstream target, and ectopic expression of MT2P1-RNA could strongly reduce the level of miR-15b-5p. Meanwhile, the degradation of MT2A mRNA induced by miR-15b-5p was also observed along with the confirmation of the direct interaction with MT2A mRNA. We prompt an axis of MT2P1-RNA/miR-15b-5p/MT2A which may regulate HCC growth through the ceRNA effect. Limited by **the lack of** experiments in vivo, we will further investigate the exact function of this axis by conducting the orthotopic transplantation model and tumorigenicity assay in animals.

Taking together the above findings, we discovered that under the control of E2F7, the transcription of pseudogene MT2P1 was suppressed. The attenuation of MT2P1-RNA weakened the ceRNA effect on depressing miR-15b-5p. Sequentially, miR-15b-5P was remarkably overexpressed in HCC cells and led to a decline of MT2A as the parental gene of MT2P1, which resulted in the progression of HCC. We believe this pseudogene-related axis might have the potential to provide innovative targets for HCC prevention and therapeutic strategy, even though there still leaves many details for investigation.

## Materials and methods

### Cell lines

Three HCC cell lines (Huh7, HepG2, and Hep3B) were applied for study, and the normal human hepatic cell LO2 was recruited as the control (Shanghai Institutes for Biological Sciences, Chinese Academy of Science, Shanghai, China). The cell lines were cultured in the RPMI 1640 medium, supplemented with 10% heat-inactivated fetal bovine serum (FBS), and were incubated at 37℃ environment temperature. The medium contained 100 ug/ml streptomycin, and 100 U/ml Penicillin, with an atmosphere of 5% CO_2_. A medium mixed with G418 (Santa Cruz Biotechnology, Inc; 400 μg/ml) was specially applied to select the transfected cells.

### Clinicopathological specimens

Paired specimens of the HCC tumor samples and the adjacent non-cancerous liver tissues were collected from the HCC patients (*N* = 95) at the Department of General Surgery, Ruijin Hospital, Shanghai Jiao Tong University School of Medicine, who received radical resection with no preoperative treatment during 2016～2020. The corresponding clinicopathologic parameters of the patients were collected including gender, age, tumor size, number of lesions, grades et.al. The informed consent for this study was approved by the Ethics Committee of Ruijin Hospital, Shanghai Jiao Tong University School of Medicine.

### Preparation of the datasets

The data of the differential gene expression for 369 liver tumors and 50 normal samples from the UCSC Xena database (https://xena.ucsc.edu/.) and also the 110 normal liver samples from the GTEx (https://www.gtexportal.org/home/.) and TCGA (https://www.cancer.gov/ccg/research/genome-sequencing/tcga) were intensively explored by using the random walk-based multi-graphic (RWMG) model algorithm developed by our team.^15^ The relative information of the pseudogene-derived transcripts was analyzed and presented in the aforementioned LCLE tools (https://datasciences123.shinyapps.io/LCLE/). Simultaneously, the starBase datasets (https://starbase.sysu.edu.cn/.) and the dreamBase (https://rna.sysu.edu.cn/dreamBase/) datasets were introduced to provide much more supplementary information on the expression and relationship of the candidate genes.

### RT-qPCR assay and immunohistochemistry assay

RNA isolation in either of the tissues or the cells was conducted according to the instructions of the TRIzol reagent (Invitrogen, USA). The first-strand cDNA was synthesized via a High-Capacity cDNA Reverse Transcription Kit (ABI, USA). The primers were prepared (Jike Biotech Company, Shanghai, China) (Supplementary Table S1). Real-time quantitative polymerase chain reaction (RT-qPCR) was conducted according to the TaqMan Gene Expression Assays protocol (ABI, USA). The relative quantification of RNA in cell lines was normalized by using GAPDH via the 2*−*Δ*CT* method. The relative quantification of miR-145-5p was measured using the mirVANATM miRNA Isolation Kit (ABI, USA). The PCR process was set by the following program: 95°C for 10 min, followed by 35 cycles of 95°C for 15 s, 60°C for the 30 s, and 72°C for 45 s.

Antibody against E2F7 was purchased from Abcam (USA). The immunohistochemistry assay was conducted using the methods we previously described.^37^ The protein expression in tissues was examined by IHC and was independently assigned to two experienced pathologists for blind examination. The samples were separated into two groups by staining intensity grade: no to low staining (0～1+) and moderate to high staining (2+～3+).

### The plasmid and cell transfection preparation

The lentiviral vector pLV (Addgene, Cambridge, USA) was applied for the extopical expression of MT2P1 and its parental gene MT2A, in the exponential phase HepG2 and Hep3B cells (JIKE Biochemistry, Shanghai, China). The lentiviral vectors pLKO.1 (Addgene, Cambridge, USA) containing shRNA was transfected into the HCC cells to deplete the expression of E2F7. For the two-way regulation of miR-15b-5p in HCC cells, either the mimic or the siRNAs was used for transfection. The rescue experiments were sequentially conducted in *vitro*, respectively.

### Cell proliferation and cell cycle detection

The intervened HCC cells (1×10^6^) were cultured in 96-well microtiter plates triplicated and incubated at an atmosphere of 5% CO_2_ and 37°C for 5 d. Microplate computer software (BioRad Laboratories, Inc., Hercules, CA, USA) was used for the OD values following the Cell Counting Kit-8 (CCK-8) assay kit protocol (Dojindo, Tokyo, Japan). The cell proliferation curves were generated. The cells were fixed by ethanol, followed by RNase A treatment and propidium iodide staining. Flow cytometry detection was conducted using FACSCalibur (Becton Dickinson, Franklin Lakes, NJ, USA) to quantify the cell populations, respectively, at the G0/G1, S, and G2/M phases. The ModFit software (Becton-Dickinson) was used. The debris and fixation artifacts of the cells were excluded.

### Cell apoptosis analysis

The rates of cell apoptosis were calculated using PE-Annexin V Apoptosis Detection Kit I (BD Pharmingen, USA). The transfected cells were resuspended under the condition of a concentration of 1×10^6^ cells/ml using 1×Binding Buffer. FITC and PI were added into the cells according to the ratio of 5 μl per 100 μl cell suspension. The 15-min incubation in darkness was then carried out and added with 400 μl×Binding Buffer. The apoptosis rate was calculated using flow cytometric (Becton Dickinson, Franklin Lakes, NJ, USA), and both Annexin V-FITC-positive and PI-negative cells were regarded as apoptosis cells. The results were analyzed in the ModFit software e (Becton Dickinson).

### Chromatin immunoprecipitation assay

A Chromatin immunoprecipitation (ChIP) assay was introduced for the verification of the interaction between the transcription factor and the targeted genes’ promoter region. A total of 5×10^6^ cells were cultured in each 10 cm dish and subjected to the ChIP assay by applying ChIP-ITTM Kit (Active Motif). Chromatin was immunoprecipitated with 2 μg of either the transcription factor antibodies (Abcam, USA) or IgG as the negative control. The DNA was extracted and analyzed through RT-qPCR assay (Supplementary Table. S1).

### Dual-luciferase reporter assay

The potential direct binding between miR-15b-5p and MT2A was predicted and displayed using the online tool of microcosm (http://mirecords.biolead.org). A 202 bp length sequence containing the putative binding site of miR-15b-5p from the 3’-UTR of MT2A mRNA was selected for designing the mutative sequence (Supplementary Table. S2). The sequences were cloned into the pMIR-Report luciferase vector, containing firefly luciferase. The pRL-TK vector luciferase was set as a control (Promega, Madison, WI, USA). The above sets of vectors were co-transfected into the HCC cells along with the introduction of either miR-15b-5p or the controls. The luciferase activity was measured via the Dual-Glo Luciferase assay system (Promega) 48 h after the transfection. The sequence and the relative mutated sequences, containing the putative binding site of miR-15b-5p of the transcript of MT2P1 were constructed (Supplementary Table. S2).

### Statistical analysis

Statistical analysis was performed in SPSS 20.0 (https://www.ibm.com/spss). Both unpaired and paired Student’s *t*-test and Fisher’s exact test were performed to evaluate the expression level differences in different groups. The statistical significance of the difference was defined by *P*-values <0.05. Experiments were repeated three times to eliminate bias.

## Supplementary Material

Suppl. materials.docx

## Data Availability

Data available on request from the authors
