# Correction

**DOI:** 10.1080/15384047.2025.2520651

**Published:** 2025-06-14

**Article title**: Suppression of pseudogene MT2P1 transcription induced by E2F7 inhibits hepatocellular carcinoma cell proliferation and facilitates apoptosis via preserving its parental gene

**Authors**: Lu, Y., Zhang, Y., Hao, F., Wang, N., Chen,Y., & Wang, J.

**Journal**: *Cancer Biology & Therapy*

DOI: https://doi.org/10.1080/15384047.2025.2510035

The author has identified an error in Figure 4. The figure has been updated, and the author requests that the figure be replaced with the revised version provided below, as it more accurately reflects the original intentions

**Figure:**
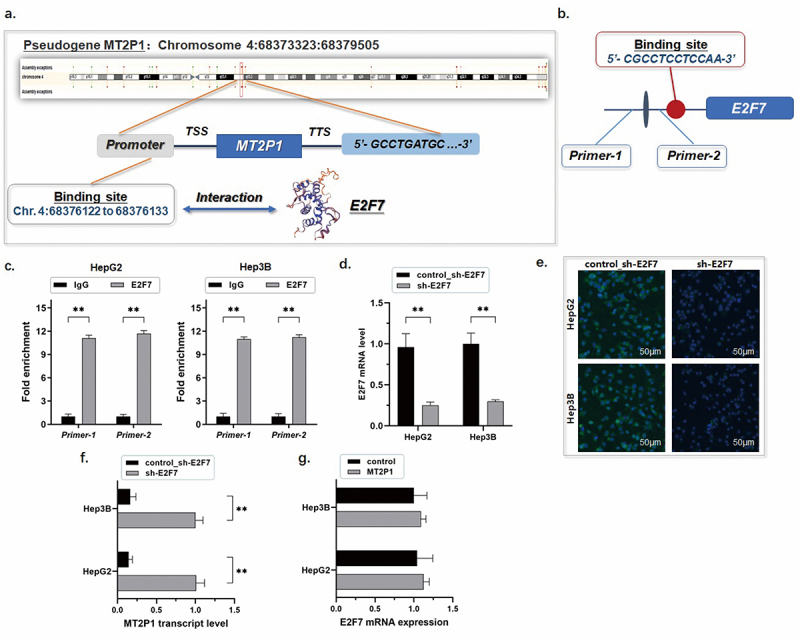

**Figure 4:**

