# Supplementary material for: Suppression of pseudogene MT2P1 transcription induced by E2F7 inhibits hepatocellular carcinoma cell proliferation and facilitates apoptosis via preserving its parental gene
Source: Cancer Biol Ther. 2025 May 23;26(1):2510035. doi: 10.1080/15384047.2025.2510035 (PMC12118385; doi:10.1080/15384047.2025.2510035)
Supplement: Suppl. materials.docx [file KCBT_A_2510035_SM0788.docx]

**Suppl. Table. 1**

**The primers for RT-qPCR assay**

| **Genes** | **Forward** | **Reverse** |
| --- | --- | --- |
| **MT2P1-RNA** | **5’-ATGGATCCCAACTGCTCCTG-3’** | **5’-TACCTAGGGTTGACGAGGAC-3’** |
| **E2F7** | **5’-GCAGTGGTTGTTTCTGTCAGG-3’** | **5’-CTGGTCAGTGTAGGGCACA**  **-3’** |
| **MT2A** | **5’-CAACCTGTCCCGACTCTAGC-3’** | **5’-TAGCAAACGGTCACGGTCAG-3’** |
| **ChIP assay**  **Primer-1** | **5’-GGCTGTTGAATGCAAGAAGGT-3’** | **5’-TACAACCTGTCCCGACTCCA-3’** |
| **ChIP assay**  **Primer-2** | **5’-TCACCATATTAAGACAAGACCCTG-3’** | **5’-CTCCAGCCGCCTCTTCAG-3’** |
|  |  |  |

**Suppl. Table. 2**

**The selected sequence of the predicted miR-15b-5p binding site of the 3’-UTR of MT2A mRNA and the MT2P1-RNA, along with the relative mutated sequences**

| **Genes** | **Sequence including the binding site**  **(202 bp)** | **Relative mutated sequence** |
| --- | --- | --- |
| **MT2A mRNA**  **3’-UTR** | 5’-aaatgcacctcctgcaagaaaagctgctgctcctgctgccctgtgggctgtgccaagtgtgcccagggctgcatctgcaaaggggcgtcggacaagtgcagctgctgcgcctgatgctgggacagccccgctcccagatgtaaagaacgcgacttccacaaacctggattttttatgtacaaccctgaccgtgaccgtttgc-3’ | 5’-aattgctcgtgcaggatgtataccaggtccacgtccaggcgtctcgccagaggctactctccgctgcggtccttgtcctatgcgccctggcagatgaggaccaggtccccgtcaaggtcgcagaccgcgggtgcgacaagaatacatccccagtacgagatagcagcaatatatttcttctagcgtcagcctcagcctatgc-3’ |
| **MT2P1- mRNA** | 5’-ggcatcaccctccacctagtggcatccctctccatctagtggcaagataggcgaattgaaagcaagtgccaaagaagaaatagtctgatactgaaatgttgaacaaaagtttaaaaagacaacagtattgaccacgcctcctccaagtcccagcgagcccgtgtacaacctgtcccgactccagccgcctcttcagctcgcc-3’ | 5’-cggaactcgcacgagcaactcggaacgcacacgaacaactcggatgtttgcccaatagtatggatgaggctatgaacataaactgtcaaagtcataagatcatctatactattatatgtctagactttagtcgagggcacgtgctactgcgacccaccgcctcttctagcagacgccagtgctggcccgtctactggtgggc-3’ |

**Supplementary Figures**

**Suppl. Fig. 1**


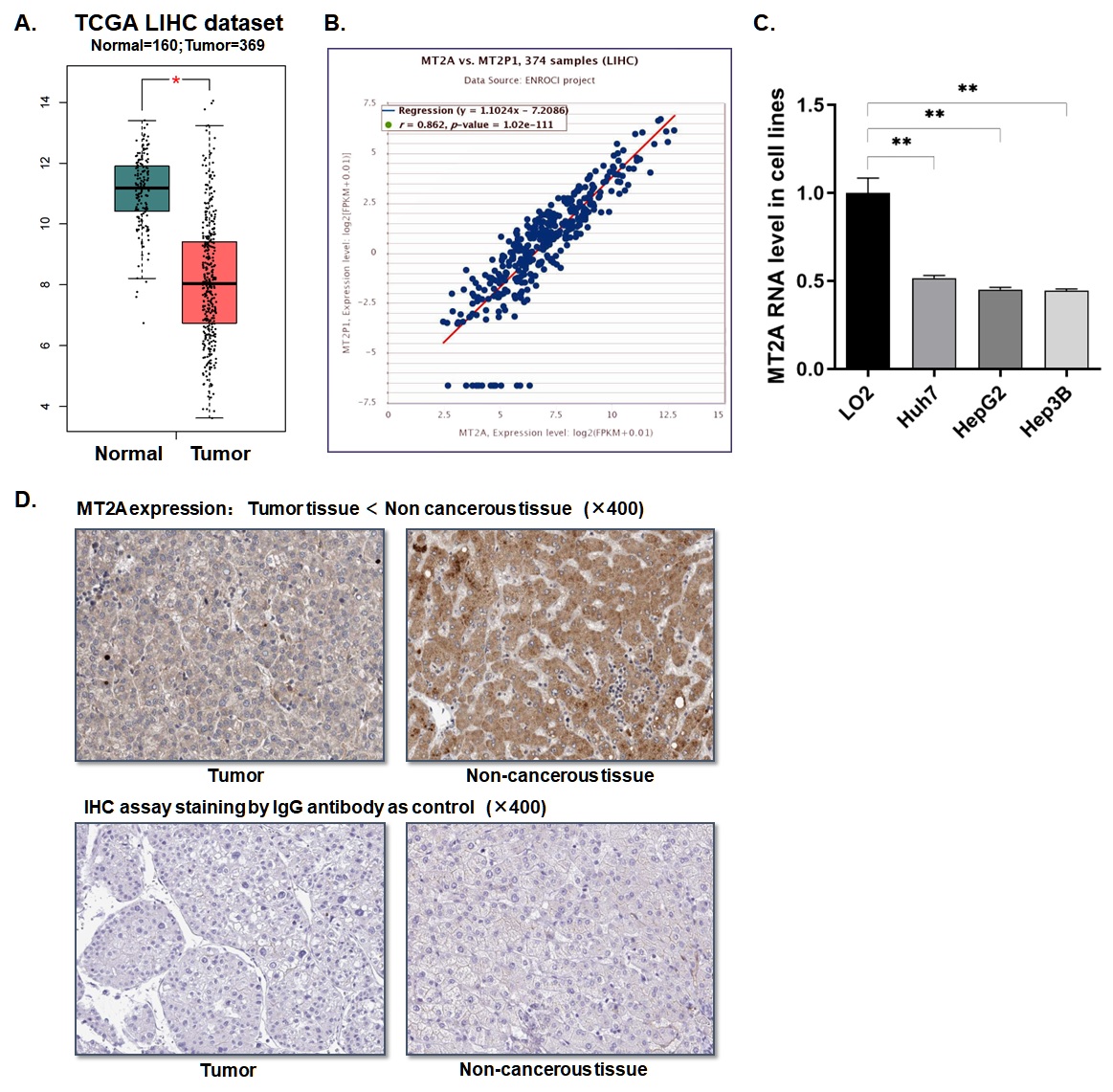


**Suppl. Fig. 1. MT2A expression profile**

**A．**According to the analysis of the TCGA database, the parental gene MT2A was also significantly decreased in HCC tumor tissues (*P<0.05). **B.** According to the analysis of the starBase online database, MT2A expression was positively correlated with the pseudogene-derived MT2P1-RNA (P=1.02e-111, R=0.862). **C.** According to the result from the RT-qPCR assay, MT2A was significantly decreased in HCC cell lines, compared with the LO2 cells (**P<0.01). **D.** Representative graph of immunohistochemistry analysis (400🞩) of the HCC cases. Specimens-stained IgG anti-body was regarded as the control. MT2A expression in tumor specimens was significantly lower than in adjacent non-cancerous tissues.

**Suppl. Fig. 2**


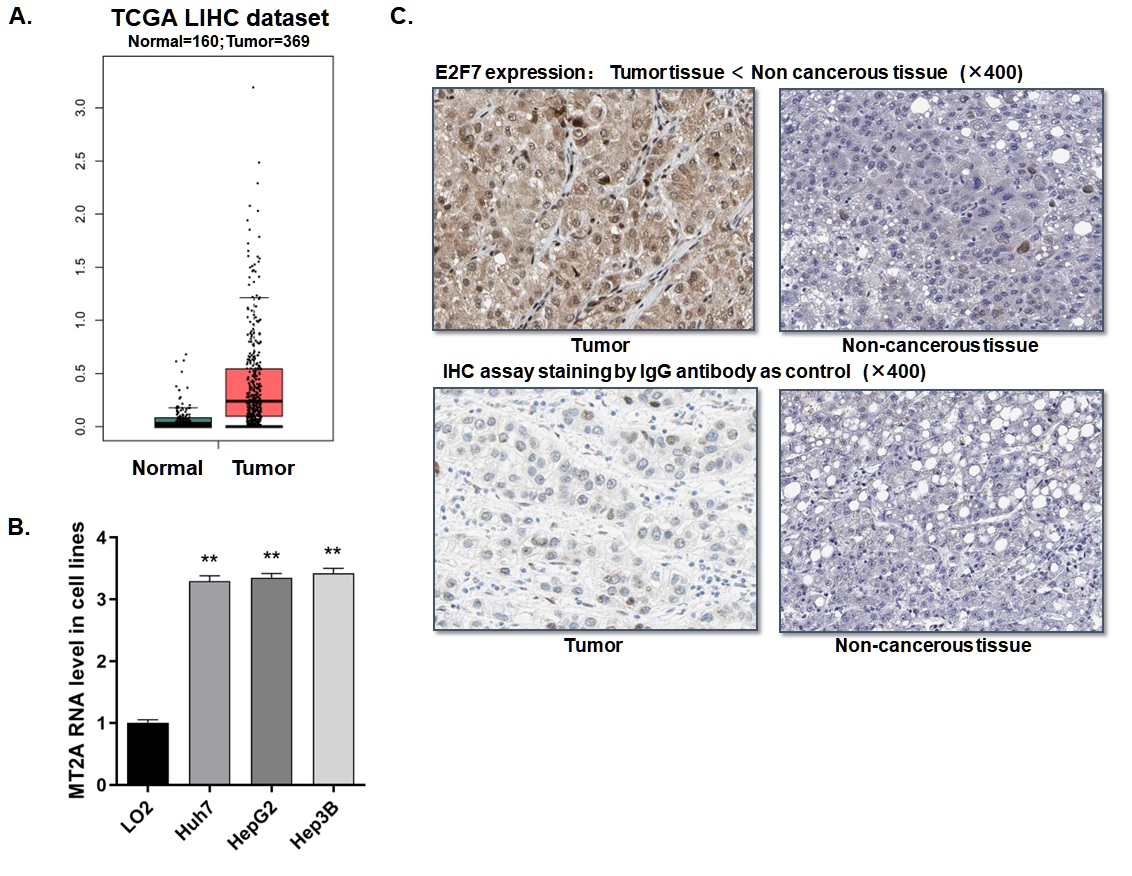


**Suppl. Fig. 2. MiR-383-5p expression profile and relationship with HCC cell proliferation**

**A.** E2F7 expression profile in HCC from the TCGA database (*P＜0.05). **B.** E2F7 mRNA levels were significantly up-regulated in the HCC cell lines, compared with the LO2 cells (**P<0.01). **C.** Representative graph of immunohistochemistry analysis (400🞩) of the HCC cases. Specimens-stained IgG anti-body was regarded as the control. E2F7 expression in tumor specimens was significantly lower than in adjacent non-cancerous tissues.
